# Supplementary material for: A modeling framework for determining modulation of neural-level tuning from non-invasive human fMRI data
Source: Commun Biol. 2022 Nov 14;5:1244. doi: 10.1038/s42003-022-04000-9 (PMC9663541; doi:10.1038/s42003-022-04000-9)
Supplement: Supplementary file 3 — Reporting Summary [file 42003_2022_4000_MOESM3_ESM.pdf]

nature research

Corresponding author(s): Patrick Sadil

Last updated by author(s): Jul 28, 2022

Reporting Summary

Nature Research wishes to improve the reproducibility of the work that we publish. This form provides structure for consistency and transparency in reporting. For further information on Nature Research policies, see our Editorial Policies and the Editorial Policy Checklist.

Statistics

For all statistical analyses, confirm that the following items are present in the figure legend, table legend, main text, or Methods section.

n/a	Confirmed	
☐	☒	The exact sample size (*n*) for each experimental group/condition, given as a discrete number and unit of measurement
☐	☒	A statement on whether measurements were taken from distinct samples or whether the same sample was measured repeatedly
☐	☒	The statistical test(s) used AND whether they are one- or two-sided<br>*Only common tests should be described solely by name; describe more complex techniques in the Methods section.*
☐	☒	A description of all covariates tested
☐	☒	A description of any assumptions or corrections, such as tests of normality and adjustment for multiple comparisons
☐	☒	A full description of the statistical parameters including central tendency (e.g. means) or other basic estimates (e.g. regression coefficient) AND variation (e.g. standard deviation) or associated estimates of uncertainty (e.g. confidence intervals)
☐	☒	For null hypothesis testing, the test statistic (e.g. F, t, r) with confidence intervals, effect sizes, degrees of freedom and P value noted<br>*Give P values as exact values whenever suitable.*
☐	☒	For Bayesian analysis, information on the choice of priors and Markov chain Monte Carlo settings
☐	☒	For hierarchical and complex designs, identification of the appropriate level for tests and full reporting of outcomes
☐	☒	Estimates of effect sizes (e.g. Cohen's d, Pearson's r), indicating how they were calculated

Our web collection on statistics for biologists contains articles on many of the points above.

Software and code

Policy information about availability of computer code

Data collection	The experiment was designed using the Psychophysics toolbox (Version 3.0.14) and custom MATLAB code (2018b, MathWorks). Eye-tracking data were recorded using the Eyelink Toolbox extension to Psychtoolbox, as distributed in version 3.0.14.
Data analysis	All models were estimated with R (R Core Team) and its interface (RStan, 2.18.2) to the Stan language. Additionally, several R packages were used. Those packages were listed in an renv file in the osf repository containing the code (see code availability statement).

For manuscripts utilizing custom algorithms or software that are central to the research but not yet described in published literature, software must be made available to editors and reviewers. We strongly encourage code deposition in a community repository (e.g. GitHub). See the Nature Research guidelines for submitting code & software for further information.

Data

Policy information about availability of data

All manuscripts must include a data availability statement. This statement should provide the following information, where applicable:

- Accession codes, unique identifiers, or web links for publicly available datasets
- A list of figures that have associated raw data
- A description of any restrictions on data availability

The imaging and behavioral data that support the findings of this study are available as a repository on the Open Science Framework (10.17605/OSF.IO/93YNM).

Field-specific reporting

Please select the one below that is the best fit for your research. If you are not sure, read the appropriate sections before making your selection.

☒ Life sciences ☐ Behavioural & social sciences ☐ Ecological, evolutionary & environmental sciences

For a reference copy of the document with all sections, see nature.com/documents/nr-reporting-summary-flat.pdf

Life sciences study design

All studies must disclose on these points even when the disclosure is negative.

Sample size	No sample-size calculation was performed in advance of data collection. However, our model recovery simulation studies (both data-uninformed and data-informed) are consistent with the acquired sample size being sufficient. That is, the simulation studies indicated that our sample size provided enough data to determine which of two types of modulation were most likely to have generated the (simulated) data.
Data exclusions	One participant completed a single session but exhibited substantial motion; their data were excluded from all analyses.
Replication	We assessed the reproducibility of our findings with a data-informed simulation. Synthetic datasets were generated from the posterior distributions of the multiplicative and additive models. Next, the models were applied to these synthetic data, keeping in mind the "ground truth", data-generating model. Across replications of synthetic datasets, the best fitting models were consistently the ground-truth models.
Randomization	We used a within-subjects design; all participants experienced the same experimental conditions.
Blinding	Blinding was not relevant for this study. The two primary conditions (high and low stimulus contrast) are easily identified in the data.

Reporting for specific materials, systems and methods

We require information from authors about some types of materials, experimental systems and methods used in many studies. Here, indicate whether each material, system or method listed is relevant to your study. If you are not sure if a list item applies to your research, read the appropriate section before selecting a response.

Materials & experimental systems

n/a	Involved in the study	
☒	☐	Antibodies
☒	☐	Eukaryotic cell lines
☒	☐	Palaeontology and archaeology
☒	☐	Animals and other organisms
☐	☒	Human research participants
☒	☐	Clinical data
☒	☐	Dual use research of concern

Methods

n/a	Involved in the study	
☒	☐	ChIP-seq
☒	☐	Flow cytometry
☐	☒	MRI-based neuroimaging

Human research participants

Policy information about studies involving human research participants

Population characteristics	The experiment involved seven participants (22 – 31 years old; 3 female, 2 did not report).
Recruitment	Subjects for fMRI experiments were recruited via three primary methods: (1) emails sent out via the University of Massachusetts Department of Psychological and Brain Sciences, to the graduate and undergraduate student populations, and to postdocs, faculty and staff, stating that paid volunteers were being sought for fMRI studies; (2) flyers posted in the Department of Psychological and Brain Sciences, the library and dorms at the university, local venues in the surrounding area, stating that paid volunteers are being sought for fMRI studies; (3) advertisements posted on Craigslist and appropriate Facebook pages, e.g., the Facebook page of the PBS Department. Some volunteers were recruited through word of mouth; all of the same information was provided to these word-of-mouth subjects.
Ethics oversight	The procedure was approved by the University of Massachusetts Institutional Review Board.

Note that full information on the approval of the study protocol must also be provided in the manuscript.

Magnetic resonance imaging

Experimental design

Design type: event-related

Design specifications:

For the main experiment, participants completed 18 functional runs across three sessions. During each run, oriented grayscale gratings were presented twice at each of two levels of contrast (eight orientations at 50% or 100% Michelson contrast in all runs for six out of seven participants, eight orientations at 20% or 80% Michelson contrast in 12 runs for one participant, and seven orientations at 20% or 80% Michelson contrast in that participant's remaining 6 runs). Grating parameters replicated those of Rademaker et al.56. Gratings (spatial frequency of 2 cycles per degree) were masked with annuli (1.2° inner and 7° outer radii). The annulus edges were smoothed with an isotropic 2D Gaussian kernel (1° kernel, 0.5° standard deviation). Throughout each run, a magenta fixation dot was presented in the center of the screen (0.2°, RGB: 0.7843, 0, 0.8886).

In each trial, a counterphasing (5 Hz) grating was presented for five seconds. In the middle three seconds of each trial, the spatial frequency of the grating either increased or decreased (1 cycle per degree) for 200 ms. Participants were instructed to indicate via button press the direction of change as soon as they noticed it. Per run, gratings were presented at multiple orientations, twice at each combination of orientation and contrast. In most runs, there were eight orientations, but in one session of one participant (totaling six runs), only seven orientations were presented. Inter-stimulus intervals ranged from 8000 – 12000 ms in steps of 200 ms. A five-second fixation period preceded the first trial, and a fifteen-second fixation period succeeded the final trial. Each run lasted 490 seconds.

Additionally, we mapped a circular area of the visual field, of radius 8° centered on a central fixation point. pRF mapping scans followed the protocol of Benson et al.57. Briefly, natural images58 were overlaid on pink noise and viewed through a series of circular apertures (8° radius). Within one run per session, the apertures enabled view of either moving bars (2°) or rotating wedges (1/4 aperture) and rings that expanded and contracted (see stimulus software for details). In bar runs, a bar traversed the central region in cycles. During each cycle, the bar was visible for 28 seconds, followed by a 4-second blank period. The bar moved in one of eight directions (east, north, west, south, northeast, northwest, southwest, or southeast, in that order). A 16-second blank period preceded the first cycle, a 12-second blank period followed the fourth cycle, and there was a 16-second blank period at the end of all cycles (300 seconds in total).

In the second pRF scan, the apertures were either wedges that rotated clockwise or counterclockwise, or they were rings that expanded or contracted. These runs started with a 16-second blank period, followed by two, 32-second cycles of a counterclockwise rotating wedge, two 28-seconds of expanding rings (each followed by a 4-second blank period), two 32-second clockwise wedge rotations, and two 28-second cycles of contracting rings (followed by 4 and 26 seconds of blank, respectively). The total run time was 300 seconds.

Behavioral performance measures:

Behavioral performance was monitored in the main functional run. Participants were instructed to indicate via button press the direction of spatial frequency change as soon as they noticed it. Average accuracy for the spatial frequency change detection task was 72% and 74% for low and high-contrast gratings, respectively ($p = 0.13$). Additionally, eye-tracking was used to assess whether participants maintained adequate fixation in the scanner: over 90% of all participants' fixations ended within 2° of the location of the run's average fixation (range: 93–99%)

Throughout the pRF scans, the color of a central fixation dot (0.3°) changed between black, white, and red. Participants were instructed to monitor the color of the fixation dot and press a button when the dot turned red. To help participants maintain fixation, a circular fixation grid was presented throughout.

Acquisition

Imaging type(s): functional, structural

Field strength: 3

Sequence & imaging parameters:

MRI data were collected on a 3T Siemens Skyra scanner with a 64-channel head coil. In each of the three sessions we collected field-mapping scans, functional scans, and a T1-weighted anatomical scan (MPRAGE, FOV 256 × 256, 1 mm isotropic, TE 2.13 ms, Flip Angle 9°). The anatomical scan was used to align field-mapping and functional images parallel to the calcarine sulcus. Gradient recall echo scans estimated the magnetic field. The pRF and primary functional data were collected with the same scan parameters (TR 1000 ms, TE 31 ms, flip angle 64°, FOV 94 × 94, 2.2 mm isotropic, interleaved acquisition, no slice gap, Multiband Acceleration Factor 4). To aid alignment of functional and anatomical images, single-band reference images were collected before each functional run for all but three participants (TR 8000 ms, TE 65.4 ms, flip angle 90°, FOV 94 × 94, 2.2 mm isotropic, interleaved acquisition, no slice gap).

Area of acquisition: The anatomical scan was used to align field-mapping and functional images parallel to the calcarine sulcus.

Diffusion MRI: ☐ Used ☒ Not used

Preprocessing

Preprocessing software: Preprocessing of images was performed with fMRIPrep 1.4.0, which relies on Nipype 1.2.0 and Nilearn 0.5.2

Preprocessing software

[RRID:SCR_001362]. The following text is a lightly edited version of the textual description of the preprocessing performed by fMRIPrep.

Anatomical Data. The T1-weighted (T1w) images were corrected for intensity non-uniformity (INU) with N4BiasFieldCorrection, distributed with ANTs 2.2.0. The T1w images were then skull-stripped with a Nipype implementation of the antsBrainExtraction.sh workflow (from ANTs), using OASIS30ANTs as target template. A T1w-reference map was computed after registration of the individual T1w images (after INU-correction) using mri_robust_template (FreeSurfer 6.0.1, RRID:SCR 001847). Brain surfaces were reconstructed using recon-all, and the brain mask estimated previously was refined with a custom variation of the method to reconcile ANTs-derived and FreeSurfer-derived segmentations of the cortical gray-matter of Mindboggle (RRID:SCR 002438). Brain tissue segmentation of cerebrospinal fluid (CSF), whit
e-matter (WM) and gray-matter (GM) was performed on the brain-extracted T1w using fast (FSL 5.0.9, RRID:SCR 002823). Functional Data. For each of the functional runs, the following preprocessing was performed. First, a reference volume and its skull-stripped version were generated using a custom methodology of fMRIPrep. A deformation field to correct for susceptibility distortions was estimated based on a field map that was co-registered to the BOLD reference, using a custom workflow of fMRIPrep derived from D. Greve's epidewarp.fsl script (www.nmr.mgh.harvard.edu/~greve/fbirn/b0/epidewarp.fsl) and further improvements of Human Connectome Project Pipelines. Based on the estimated susceptibility distortion, an unwarped BOLD reference was calculated for a more accurate co-registration with the anatomical reference. The BOLD reference was then co-registered to the T1w reference using bbregister (FreeSurfer) which implements boundary-based registration. Co-registration was configured with nine degrees of freedom to account for distortions remaining in the BOLD reference. Head-motion parameters with respect to the BOLD reference (transformation matrices, and six corresponding rotation and translation parameters) were estimated before any spatiotemporal filtering using mcflirt (FSL 5.0.9). The BOLD time-series were resampled to surfaces on the following spaces: fsaverage and fsnative (FreeSufer). The BOLD time-series were resampled onto their original, native space by applying a single, composite transform to correct for head-motion and susceptibility distortions. These resampled BOLD time-series will be referred to as "preprocessed BOLD". A reference volume and its skull-stripped version were generated using a custom methodology of fMRIPrep. A set of physiological regressors were extracted to allow for component-based noise correction. Principal components are estimated after high-pass filtering the pre-processed BOLD time-series (using a discrete cosine filter with 128 s cut-off) for the anatomical CompCor (aCompCor). The time-series entering the CompCor analyses are derived from a mask at the intersection of subcortical regions with the union of CSF and WM masks calculated in T1w space, after their projection to the native space of each functional run (using the inverse BOLD-to-T1w transformation). Gridded (volumetric) resamplings were performed using antsApplyTransforms (ANTs), configured with Lanczos interpolation to minimize the smoothing effects of other kernels. Non-gridded (surface) resamplings were performed using mri_vol2surf (FreeSurfer).

Normalization

To reduce data smoothing, data were not normalized to a common template.

Normalization template

n/a

Noise and artifact removal

Design matrices contained both six motion (three translation and three rotation) and multiple aCompCor regressors. For each run, the number of components was determined by the broken-stick method.

Volume censoring

n/a

Statistical modeling & inference

Model type and settings

To estimate voxel-wise responses to each orientation, a general linear model (GLM) was fit using SPM12 (version 7487, RRID:SCR 007037) to the time-series of each voxel during each orientation run. Fitting the GLM can be viewed as a preprocessing step to reduce the dimensionality of the data; the method presented here could be configured to run on the raw timeseries, but working with beta weights of a GLM rather than the raw timeseries drastically reduced the computational requirements of the Bayesian estimation. Prior to fitting the GLM, each voxel's timeseries was converted into a percent signal change, relative to the average signal within a run (across voxels). Design matrices were convolved with the canonical hemodynamic response function, parameterized with the SPM12 defaults, and additionally contained both six motion (three translation and three rotation) and multiple aCompCor regressors. For each run, the number of components was determined by the broken-stick method.

Effect(s) tested

n/a

Specify type of analysis: ☐ Whole brain ☒ ROI-based ☐ Both

Anatomical location(s)

We restricted the analyses to only striate voxels whose population receptive fields (pRFs) did not overlap with the stimulus edges. We estimated the pRFs of each voxel with standard methods. First, the preprocessed functional data for the pRF scans were converted into percent mean signal change within a run (across voxels). The compressive spatial summation model was fit to each voxel using analyzePRF HCP7TRET 52,57. Following Benson et al., the compressive exponent of this model was set to 0.05. The resulting pRF parameters were combined with an anatomical prior for a Bayesian estimation of the parameters (neuropythy 0.94)62. Only the parameters of voxels for which the pRF explained more than 10% of the variance of the run were used as empirical parameters for Bayesian estimation; the remaining voxels' posterior pRF parameters were determined entirely by the prior.

The resulting pRF parameters determined whether a voxel would be retained for analyses. The pRF resembles an isotropic, bivariate Gaussian. The three pRF sessions were analyzed separately, resulting in three sets of three pRF parameters per voxel. Within a set of parameters, two indicate the center location of the pRF, and the third determines its size – the standard deviation of the Gaussian. A voxel was retained only if a circle centered on its pRF with radius equal to two standard deviations was entirely contained by the grating stimulus in each of the three sessions.

Statistic type for inference
(See Eklund et al. 2016)

The GLM was fit to each voxel separately. The Bayesian model used the estimated beta-weights hierarchically, as described in the manuscript and supplementary materials.

Correction

n/a

Models & analysis

n/a	Involved in the study
☒	☐ Functional and/or effective connectivity
☒	☐ Graph analysis
☒	☐ Multivariate modeling or predictive analysis
